# Comparison of Progesterone Concentrations in Saliva and Blood in Intact Bitches

**DOI:** 10.1111/rda.70333

**Published:** 2026-11-03

**Authors:** Rebekka Engel, Jessica Cremer, Eva‐Maria Bauer, Laura Meder, Maike Schroers, Yury Zablotski, Beate Walter

**Affiliations:** ^1^ Small Animal Clinic Ludwig‐Maximilians University Munich Munich Germany; ^2^ Clinic for Ruminants With Ambulatory and Herd Health Services Ludwig‐Maximilians University Munich Oberschleissheim Germany

**Keywords:** chemiluminescence immunoassay, dog, non‐invasive method, ovulation timing, steroid hormones

## Abstract

Accurate determination of the optimal mating time is essential in canine breeding management, with serum progesterone measurement serving as a key diagnostic tool. However, repeated blood sampling causes stress in dogs, motivating research into non‐invasive alternatives. Salivary diagnostics have shown promise for other hormones in dogs, but progesterone detection in saliva has not yet been reliably established in this species. The objective of the present study was to measure progesterone in canine saliva as described in women and to ascertain whether these values correlate with serum progesterone concentrations. A total of 34 paired blood and saliva samples were collected from 21 intact female dogs with a mean age of 3.5 years (range 1–10 years) and a mean weight of 26.1 kg (range 7.5–58 kg). Samples were taken at different stages of the reproductive cycle to ensure a broad range of progesterone concentrations and analysed using a chemiluminescence immunoassay (CLIA; IMMULITE 2000 XPi, Siemens). Salivary progesterone was detectable in 30 of 34 samples. A significant positive correlation was found between saliva and serum progesterone concentrations (*p* = 0.005, marginal *R* ^2^ = 0.18, conditional *R* ^2^ = 0.68), accompanied by high individual variation. Although progesterone can be measured in canine saliva using CLIA, it is currently not reliable for clinical use. Salivary diagnostics remain a promising non‐invasive approach, but further methodological development and larger sample sizes are needed before salivary progesterone can be applied to oestrous cycle monitoring in bitches.

## 1 Introduction

Successful breeding management requires determining the optimal mating time, particularly in artificial insemination (Steckler et al. 2013; Thomassen et al. 2006). Measuring serum progesterone in combination with other tests is one of the most important diagnostic tools for this purpose (Conley et al. 2023; Hewitt and England 2000). Nevertheless, the method necessitates recurrent veterinary consultations and blood sampling, which can induce stress and discomfort for the dog (Doring et al. 2009; Hekman et al. 2012; Stanford 1981).

With the aim to reduce stress factors in veterinary medicine, saliva diagnostics have already been used extensively in studies as a non‐invasive diagnostic methods for other parameters such as cortisol, vasopressin and oxytocin in dogs (Hekman et al. 2012; MacLean et al. 2018; Pirrone et al. 2019; Schroers, Juhasz, et al. 2024; Schroers, Zablotski, and Meyer‐Lindenberg 2024).

In human medicine, saliva‐based diagnostics have proven to be an effective, non‐invasive diagnostic alternative to blood samples and have been successfully established in numerous studies for monitoring estradiol, progesterone and cortisol (Gröschl et al. 2001; Lu et al. 1997; Meulenberg and Hofman 1989; Reynolds et al. 2018; Sakkas et al. 2021). A key advantage is that the samples could be collected at home, allowing repeated sampling for monitoring the hormone rhythm. Previous studies comparing salivary and serum progesterone via radioimmunoassay (RIA) demonstrated strong positive correlations with coefficients up to 0.9 (Gröschl et al. 2001; Lu et al. 1997).

To our knowledge only one study has previously investigated salivary diagnostics for determining optimal mating periods in dogs. However, this study was based on saliva crystallisation rather than progesterone measurement. The authors evaluated the saliva of nine female beagles during oestrus. The results obtained demonstrated a sensitivity of only 40.6%, accompanied by a specificity of 86.1% for detection of ovulation, which indicates that crystallisation patterns are not sufficiently reliable for this purpose in bitches (Pardo‐Carmona et al. 2010). A study in alpacas investigated the potential of progesterone in saliva for pregnancy diagnosis. However, this method was not found to be reliable too (Volkery et al. 2012).

Other studies on non‐invasive methods of measuring progesterone have been conducted more frequently with different outcomes. In a study of intact pregnant and non‐pregnant female dogs, a significant correlation between progesterone measurements in faeces and serum was demonstrated (Gudermuth et al. 1998). In a subsequent study, this finding was corroborated in Eurasian lynxes. However, it became evident that species‐specific excretion pathways and metabolites should be considered when evaluating these results (Dehnhard et al. 2008). Furthermore, the successful analysis of progesterone levels in urine has been demonstrated in lynxes and alpacas (Braun et al. 2009; Volkery et al. 2012), and measurement of progesterone in milk has been well‐established in cows (Bruinje et al. 2019; Hoffmann et al. 1976; Schiavo et al. 1975). In recent studies on pregnancy testing in alpacas and lamas, a significant correlation between progesterone levels in the blood and milk has also been demonstrated (Leisen et al. 2025; Volkery et al. 2012).

The objective of the present study was to measure progesterone in saliva using a conventional method, in this case chemiluminescence immunoassay (CLIA), and to ascertain whether these values correlate with serum progesterone concentrations. Additionally, the influence of body weight or salivation level on salivary progesterone concentrations was examined.

## 2 Materials and Methodes

The present study was approved by the Ethics Committee of the Faculty of Veterinary Medicine, LMU Munich (approval no. 420‐31‐10‐24, confirmed 31 October 2024). Sample collection was confirmed by the institutional animal welfare officer not to constitute an animal experiment under Directive 2010/63/EU and the German Animal Welfare Act.

### 2.1 Animals and Blood Sample Collection

34 blood and saliva samples were collected from 21 intact female dogs weighing over 5 km which were presented to our clinic. Physical examination was unaltered in all dogs. Breed, age, and body weight at the time of examination were recorded.

To ensure a broad range of progesterone concentrations, samples were collected at different time points of the reproductive cycle as part of the routine diagnostic procedure for pre‐breeding examination or for pregnancy monitoring between Days 25 and 35 post‐ovulation (p.o.). Blood samples were collected by puncturing the vena saphena. The samples were then centrifuged and the supernatant pipetted into an Eppendorf tube. Which were stored at −80°C until measurement. Saliva samples were obtained by a saliva swab (SalivaBio Children's Swab, Salimetrics LLC, USA) placed in the oral cavity and held in position by hand for approximately 2 min. Ideally, the dog chewed gently on the swab to stimulate saliva production. The swabs were then placed in designated storage tubes (Swab Storage Tube, Salimetrics LLC, USA) and centrifuged. The expressed saliva (minimum 200 μL per sample) was transferred to an Eppendorf tube and stored at −80°C until analysis.

### 2.2 Hormone Analyses

All frozen samples were subsequently submitted to a commercial lab for analysis using a chemiluminescence immunoassay (IMMULITE 2000 XPi, Siemens). Both blood and salivary samples were measured in three separate batches in singlet. Blood samples were analysed according to the manufacturer's instructions. As no commercial saliva‐specific progesterone assay was available for this platform, salivary progesterone was analysed using the same serum assay protocol. Intra‐assay coefficient of variation (CV) of 3.72%–4.77% and an inter‐assay coefficient of variation (CV) of 2.72%–3.32% are described for canine serum (Hinderer et al. 2021).

### 2.3 Statistical Analysis

The statistical analysis was performed using the statistical software R (R version 4.5.0 (2025‐04‐11)). A priori power analysis was conducted for a two‐sided correlation test using the pwr.r.test function in R statistical software with an expected effect size of *r* = 0.50, significance level *α* = 0.05 and desired power 1 − *β* = 0.80. This analysis indicated that 28.2 sample pairs are required. To account for potential attrition during data collection, a 20% buffer was added. Multiplying the estimated sample size by 1.2 gave 33.84, which was rounded up to 34 sample pairs.

Data analysis was performed using R 4.5.0 (2025‐04‐11). Due to the presence of repeated measures, linear mixed effects models with individual animal as a random effect were chosen for analysis. The normality of residuals was checked visually via QQ‐plots and via the Shapiro–Wilk normality test. The heteroscedasticity (constancy of error variance) was also checked visually and via the Breusch–Pagan test. Since assumptions were satisfied, linear mixed effects models were used (R package—lmer). Results with a *p*‐value < 0.05 were considered statistically significant.

In order to identify possible factors influencing progesterone concentration in saliva, saliva volume and body weight were examined as potential covariates. Therefore, the dogs were divided into two groups due to saliva volume and body weight by the sample median, which was 0.29 mL for saliva and 20.0 kg for body weight. These covariate analyses were conducted as secondary, exploratory analyses and were not included in the primary sample size calculation, which was based on the correlation between blood and saliva progesterone concentrations.

## 3 Results

The 21 female canines were of 15 different breeds with a mean age of 3.5 years (range 1.0–10.0 years) and a mean weight of 26.1 kg (range 7.5–58.0 kg).

The 34 sample‐pairs consisted of six sample‐pairs collected from bitches in the late proestrus to preovulatory oestrus exhibiting low progesterone levels below 15 nmol/L in blood and 17 collected from bitches after ovulation and during pregnancy displaying high progesterone levels above 15 nmol/L. Furthermore, a total of 11 sample‐pairs were collected during the course of progesterone progression. In four bitches, two sample‐pairs were collected and in one bitch, three sample‐pairs were collected at intervals ranging from one to 5 days (Table 1).

**TABLE 1:** Number of bitches and sample pairs by reproductive stage.

Reproductive stage	Blood progesterone (nmol/l)	Bitches (*n*)	Sample pairs (*n*)
Proestrus to preovulatory oestrus	1.33–9.0	6	6
Post ovulation, metestrus/pregnancy	> 15.0	14	17
Repeated sampling across progesterone progression^a^		5	11
Total		21^b^	34

^a^ Sample pairs obtain at intervals of one stage to 5 days within the same productive cycle to characterise the progesterone rise.

^b^ The bites (*n*) column sums to 25 rather than 21 because three animals were sampled during two different reproductive cycles up to 6 months later. These animals are therefore counted only once in this category above. The total number of distinct animals contributing to the study was 21.

Serum progesterone levels ranged from 1.33 nmol/L to a maximum of 127.0 nmol/L, with a mean of 26.8 nmol/L.

The presence of progesterone was detected in most saliva samples (*n* = 30). Four samples were found to be below the detection limit of 0.16 nmol/L and were included in the statistical analysis using the detection limit value. The highest concentration of progesterone in saliva was measured at 3.21 nmol/L and the mean was 0.71 nmol/L.

There was a significant correlation (estimate = 0.01, 95% CI 0.00–0.02, *p* = 0.005; marginal *R* ^2^ = 0.18, conditional *R* ^2^ = 0.68) between saliva and serum progesterone concentrations (Figure 1). However, a high degree of inter‐individual variation and inconsistency between media was observed. In particular, the group with repeated sampling (*n* = 5), only two bitches showed a progesterone progression in the salivary samples.

**FIGURE 1:**
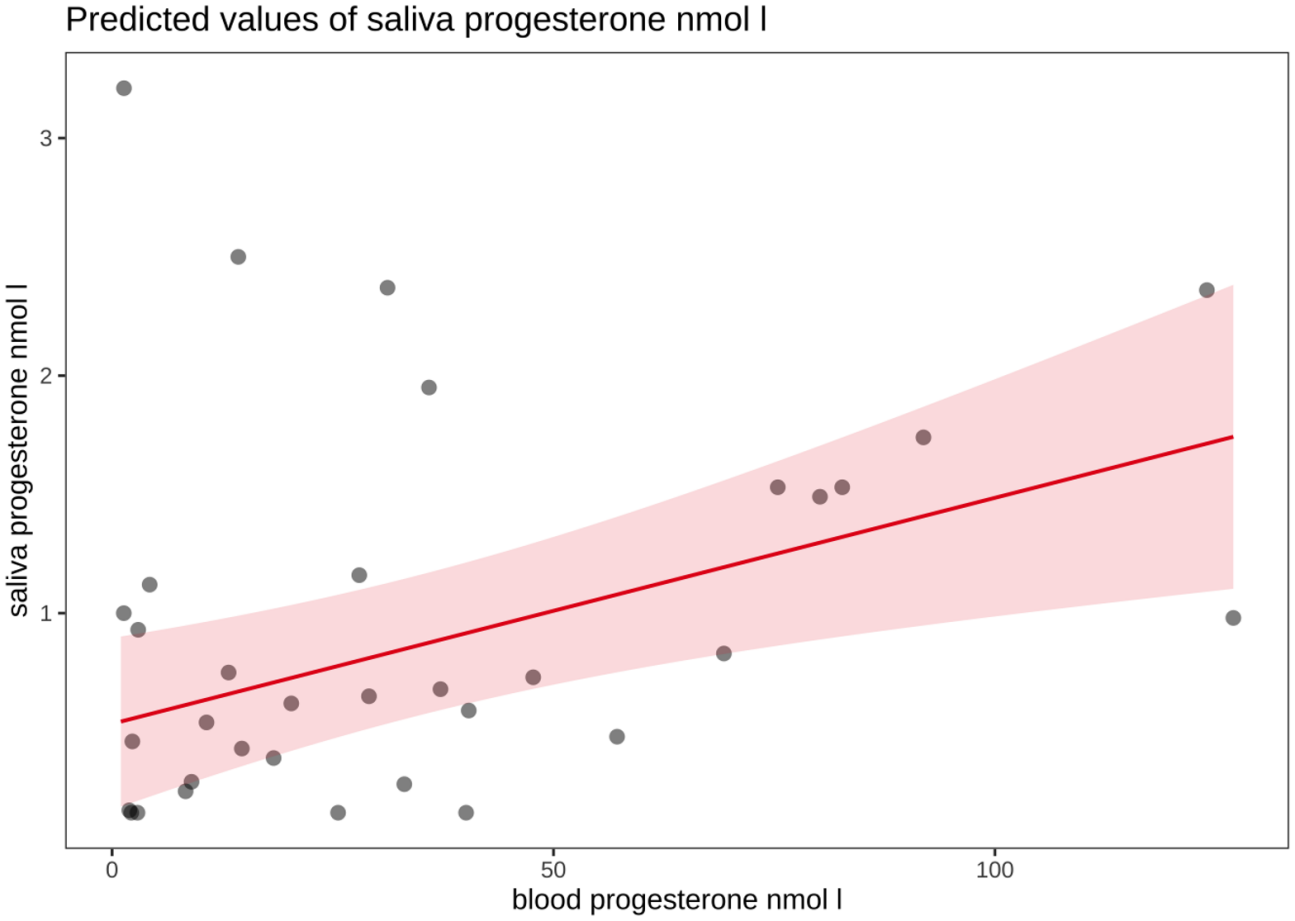
Correlation of progesterone concentrations in blood and saliva (nmol/L) in intact bitches during oestrus cycle monitoring (*n* = 34).

In order to analyse co‐factors that might influence progesterone concentration in saliva, the samples were divided into two groups for each co‐factor. To analyse the influence of salivation levels, a median split was performed on the collected saliva volumes. Dogs that produced < 0.29 mL of saliva within the 2‐min timeframe were categorised as having low salivation (*n* = 17), while those that produced 0.29 mL and more were categorised as having high salivation (*n* = 17). To analyse the influence of body weight, the dogs were categorised as having either a body weight below 20 kg (*n* = 23) or 20 kg and above (*n* = 11). The investigation revealed that saliva volume (≤ 0.29 mL; *p* = 0.0016, > 0.29 mL; *p* = 0.13) as well as weight (≤ 20 kg; *p* = 0.0061; > 20 kg; *p* = 0.256) exhibited a statistically significant influence on the correlation between serum and salivary progesterone concentrations. Smaller volumes had a significant correlation between serum and salivary progesterone as well as a lower weight.

## 4 Discussion

The study aimed to measure progesterone in saliva using a conventional method, and to investigate whether these values correlated with serum progesterone concentrations.

We detected progesterone in saliva and demonstrated a statistically significant, positive correlation between saliva and serum progesterone concentrations (*p* = 0.005). However, large variations were observed between individual animals. This was also reflected in the intraclass correlation coefficient (ICC = 0.61). The marginal *R* ^2^ (0.18) was considerably lower than the conditional *R* ^2^ (0.68). This indicates that blood progesterone alone explains only a modest proportion of the variance in saliva progesterone, whereas individual animal differences account for a substantially larger proportion.

In humans, correlation coefficients of up to 0.9 have been achieved; however, radioimmunoassay (RIA) was used in these studies to analyse serum progesterone (Gröschl et al. 2001; Lu et al. 1997; Wong et al. 1990). This method was also previously used as the gold standard for the detection of progesterone in serum (Hussein et al. 2022). However, this method is associated with high laboratory costs and risks for laboratory staff due to the radioactive isotopes used. In the meantime, other measurement methods such as CLIA and enzyme‐linked immunosorbent assay (ELISA) have established themselves as reliable alternatives (Gloria et al. 2018; Hussein et al. 2022; Schooley et al. 2019). We therefore opted for CLIA in our experiment, as correlation coefficients of 0.96 between progesterone measurements from CLIA and RIA have been demonstrated (Gloria et al. 2018).

In a further study in humans, Sakkas et al. (2021) measured salivary progesterone using enzyme‐linked immunosorbent assay (ELISA) kits. In contrast to oestradiol, only a weak correlation (*r* = 0.03) was found. The authors attributed this to daily individual fluctuations in progesterone (Delfs et al. 1994; Steinetz et al. 1990; Syrop and Hammond 1987) and abnormal behaviour of progesterone in saliva in infertile women. Our bitches were all clinically healthy; however, the presence of subclinical conditions cannot be ruled out. This could be a further cause of the low correlation, even though blood and saliva samples were always collected concurrently.

Several factors likely contribute to the observed discrepancy between blood and saliva progesterone concentrations. We found a significant interaction between blood progesterone concentration and saliva volume (*p* = 0.039), with a significant association only in dogs producing lower saliva volumes (≤ 0.29 mL; *p* = 0.0016) but not in those producing higher volumes (*p* = 0.130). This is consistent with human data showing that higher saliva flow dilutes progesterone concentration, as hormone secretion does not increase proportionally with flow rate (Zhu et al. 2021).

Other proposed explanations include differences in saliva composition between species, resulting in discrepancies between our findings and those in human studies (Hardaker et al. 2025) as the proportion of free versus protein‐bound progesterone entering saliva (Meulenberg and Hofman 1989; Wang and Knyba 1985), and local salivary metabolism of progesterone (Gröschl et al. 2001; Laine and Ojanotko 1999). These explanations remain plausible but are not supported by canine‐specific data and could not be further examined in this study.

Due to the lower sample size in this preliminary study, individual variations carried greater weight; also, the subgroup analyses for saliva volume and body weight were based on sample sizes (*n* = 17/17 and *n* = 23/11, respectively) that were not independently powered and should therefore be interpreted with caution. To evaluate the applicability of the method under real‐world conditions, we recruited study participants from dogs seen in routine clinical practice. Factors such as water and food intake prior to sample collection could therefore only be controlled to a limited extent, and for this reason, our study did not impose any restrictions on feed and water intake prior to sampling. However, these factors may also have an influence on the results. In human medical studies, participants are generally instructed not to eat or drink anything for up to 30 min prior to sample collection, and to rinse their mouths with water approximately 10 min beforehand to prevent contamination of the saliva by blood or other substances (Bolaji 1994; Sakkas et al. 2021; Schiffer et al. 2019).

Another limitation of saliva‐based diagnostics is the difficulty in obtaining sufficient sample material. In the present study, two saliva samples could not be analysed due to insufficient sample volume. Both samples were collected in summer, where more dogs tend to be slightly dehydrated. Another reason for the insufficient sample volume could be stress which leads to reduced salivation (Gholami et al. 2017; Treder‐Rochna et al. 2026).

Due to the limited sample volume and budget constraints, progesterone levels were measured in singlet rather than duplicate. Although duplicate testing is recommended to minimise intra‐assay variability and potential pipetting errors, single measurements are common practice in routine clinical settings. Nevertheless, this remains a methodological limitation of our study. Furthermore, the reported coefficients of variation refer to a validation study from Hinderer et al. (2021) in canine serum. To our knowledge, there is no corresponding data for saliva, and the limited saliva volume obtained did not permit additional replicate testing to establish this.

Salivary progesterone testing is not currently available for clinical use. However, salivary diagnostics in general, such as cortisol testing, have already been shown to offer a promising and stress‐free alternative to conventional blood‐based diagnostics in other areas. It is recommended that future research concentrate on the development of a methodology that is more sensitive yet cost‐effective. Subsequent testing of this hypothesis in larger study groups is recommended, with the objective of minimising the influence of individual variations.

## 5 Conclusion

Although progesterone can be detected in saliva and it correlates significantly with blood levels, high individual variations were observed. This means saliva levels are not currently suitable for clinical use. Nevertheless, salivary diagnostics is a promising non‐invasive method for detecting steroid hormones; however, further research into salivary progesterone is required before its use in clinical practice for the determination of a bitch's oestrous cycle can be considered.

## Author Contributions

**Rebekka Engel:** writing – original draft, conceptualization, data curation. **Jessica Cremer:** writing – review and editing, data curation. **Laura Meder:** writing – review and editing, data curation. **Eva‐Maria Bauer:** writing – review and editing, data curation. **Yury Zablotski:** writing – review and editing, visualization, methodology, formal analysis. **Maike Schroers:** writing – review and editing, conceptualization. **Beate Walter:** writing – review and editing, supervision, conceptualization.

## Conflicts of Interest

The authors declare no conflicts of interest.

## Data Availability

The data that support the findings of this study are available on request from the corresponding author.
